# Network pharmacology and molecular docking analysis on mechanisms of Tibetan Hongjingtian (*Rhodiola crenulata*) in the treatment of COVID-19

**DOI:** 10.1099/jmm.0.001374

**Published:** 2021-07-27

**Authors:** Li Wang, Yuhe Wang, Wei Yang, Xue He, Shilin Xu, Xiaoli Liu, Yongjun He, Qunying Hu, Dongya Yuan, Tianbo Jin

**Affiliations:** ^1^​ Key Laboratory of Molecular Mechanism and Intervention Research for Plateau Diseases of Tibet Autonomous Region, School of Medicine, Xizang Minzu University, Xianyang, Shaanxi 712082, PR China; ^2^​ Department of clinical laboratory, The Affiliated Hospital of Xizang Minzu University, Xianyang, Shaanxi 712082, PR China

**Keywords:** COVID-19, cytokine storm, Hongjingtian (*R. crenulata)*, molecular docking, network pharmacology

## Abstract

**Introduction:**

Coronavirus disease 2019 (COVID-19) is a highly contagious disease and ravages the world.

**Hypothesis/Gap Statement:**

We proposed that *R. crenulata* might have potential value in the treatment of COVID-19 patients by regulating the immune response and inhibiting cytokine storm.

**Aim:**

We aimed to explore the potential molecular mechanism for *Rhodiola crenulata* (*R. crenulata*)*,* against the immune regulation of COVID-19, and to provide a referenced candidate Tibetan herb (*R. crenulata*) to overcome COVID-19.

**Methodology:**

Components and targets of *R. crenulata* were retrieved from the TCMSP database. GO analysis and Kyoto Encyclopaedia of Genes and Genomes (KEGG) pathway enrichment were built by R bioconductor package to explore the potential biological effects for targets of *R. crenulata*. The *R. crenulata*-compound-target network, target pathway network and protein–protein interaction (PPI) network were constructed using Cytoscape 3.3.0. Autodock 4.2 and Discovery Studio software were applied for molecular docking.

**Result:**

Four bioactive components (quercetin, kaempferol, kaempferol-3-O-α-l-rhamnoside and tamarixetin) and 159 potential targets of *R. crenulata* were identified from the TCMSP database. The result of GO annotation and KEGG-pathway-enrichment analyses showed that target genes of *R. crenulata* were associated with inflammatory response and immune-related signalling pathways, especially IL-17 signalling pathway, and TNF signalling pathway. Targets-pathway network and PPI network showed that IL-6, IL-1B and TNF-α were considered to be hub genes. Molecular docking showed that core compound (quercetin) had a certain affinity with IL-1β, IL-6 and TNF-α.

**Conclusion:**

*R. crenulata* might play an anti-inflammatory and immunoregulatory role in the cytokine storm of COVID-19.

## Introduction

Coronavirus disease 2019 (COVID-19) is a highly contagious disease caused by severe acute respiratory syndrome coronavirus 2 (SARS-CoV-2) that ravages the world resulting in a pandemic of increasing death [1]. This novel coronavirus has rapid and extensive spread and general susceptibility of the population, which make COVID-19 be a highly contagious disease [2]. Clinical symptoms of COVID-19 patients were acute pneumonia, systemic fever, dry cough, fatigue, myalgia/arthralgia, and breathing difficulties etc., and severe cases can lead to acute respiratory distress, multiple-organ failure, and even death [3]. Some studies supported that, during the response to SARS-CoV-2, the immune dysregulation and the high level of proinflammatory cytokines could occur in some infected patients, which were called as the cytokine storm [4]. Cytokine storm causes acute respiratory distress syndrome (ARDS) or multiple-organ dysfunction, which may play an important role in the clinical deterioration of COVID-19 [5]. Therefore, effectively suppressing the cytokine storm is the key to preventing the deterioration of COVID-19 and improving the treatment success rate.

Currently, there are few effective medications for treating COVID-19. Some studies have shown that traditional Chinese Medicine (TCM) plays an important role in prevention and treatment of COVID-19. Hongjingtian (*Rhodiola*), the genus *Rhodiola* in the family *Crassulaceae*, is herbaceous perennial plants. There are 96 species of *Rhodiola* in the world and most are found in different regions of China (73 species), such as Tibet. *Rhodiola*, also known as ‘oriental god grass’ and ‘plateau ginseng’, has great medicinal value. The main chemical components of *Rhodiola* include salidroside, flavonoids, terpenoids, sterols, tannins and other compounds. The functions of *Rhodiola* might be involved in promoting blood circulation and removing blood stasis, clearing lung and relieve cough, reducing fatigue and weakness, antiviral infecting and improving immunity [6, 7]. Furthermore, modern research has shown that the comprehensive nourishing effects of the *Rhodiola* species are largely attributed to its phytochemicals, which exert anti-hypoxic, anti-viral, immune regulatory, anti-tumour, anti-fatigue, anti-depressive, and improvement of learning and memory effects [8–10]. Current investigation reveals that *Rhodiola crenulata* (*R. crenulata*) has pharmacological prevention and treatment for many diseases including influenza, sepsis, lung injury and trachea inflammation [11, 12]. *R. crenulata* might have potential value in the treatment of COVID-19 patients by regulating the immune response and inhibiting cytokine storm.

According to Flora of China, *Rhodiola crenulata* is mainly distributed in Tibet (also named Xizang), PR China [13]. *Rhodiola* grows on alpine grasslands, valley rocks or glaciers at an altitude range of 1800–5600 m and can adapt to extremely high altitude adversities, including a low temperature, hypoxia, intensive ultraviolet radiation, huge diurnal temperature differences, etc. In the present study, the bioinformatics, network pharmacology and molecular docking were used to predict their potential targets and signal pathways of Tibetan herb *R. crenulata* and to analyse the relationship of the active compounds with targets. These results are expected to help understand the potential molecular mechanism for Tibetan herb *R. crenulata* against the immune regulation of COVID-19, and to provide a referenced candidate TCM Tibetan herb to overcome COVID-19.

## Methods

### Identification of bioactive components of *R. crenulata*

The components of *R. crenulata* were retrieved from the traditional Chinese medicine systems pharmacology (TCMSP) database (http://tcmspw.com/) and previous studies [14, 15]. Oral bioavailability (OB) represents the ratio of an orally administered dose compared to unchanged drug that reaches the systemic circulation, which is one of the most significant pharmacokinetic parameters [16]. Drug-likeness (DL) is a qualitative concept to estimate the drug-ability of a molecule [17]. Substances with OB ≥30 % and DL index ≥0.18 were regarded to have high OB and drug ability. Therefore, bioactive components of candidate herbs with OB ≥30 % and DL index ≥0.18 were identified for subsequent analysis in the current study.

### Construction of *R. crenulata*-compound-target network

The target protein of bioactive components in *R. crenulata* was also retrieved from TCMSP database. Afterward, the target proteins corresponding to the compounds screened from the Pharmmapper database and PubMed database were standardized in UniProt (http://www.uniprot.org/). The targets from different databases were merged and the duplicated targets were removed. Finally, Cytoscape 3.3.0 software (http://www.cytoscape.org/) was used to construct the herb-compound-target network, which helps to understand the pharmacological mechanism of *R. crenulata*.

### Gene ontology and pathway enrichment analysis for targets of *R. crenulata*

DAVID (the Database for Annotation, Visualization and Integrated Discovery, http://david.abcc.ncifcrf.gov/) and KOBAS [Kyoto Encyclopaedia of Genes and Genomes (KEGG) Orthology Based Annotation System, https://www.biostars.org/p/200126/] were utilized for retrieving information about functional annotation of genes. Gene ontology (GO) analysis and KEGG-pathway enrichment were built by R bioconductor package to explore the potential biological effects for targets of *R. crenulata*. GO terminology was annotated including biological process (BP), cellular component (CC) and molecular function (MF) categories. KEGG-pathway database (https://www.kegg.jp/kegg/) was applied for the targets mapped to the pathway. The target-pathway/function network was constructed using Cytoscape 3.3.0 to identify the relationships of *R. crenulata* targets with the involved pathways obtained through enrichment analysis.

### Protein–protein interaction analysis

Screening for the immunity and inflammation cytokines in COVID-19 among *R. crenulata* targets were performed. The STRING database (http://string-db.org/) is a search tool for retrieval of interacting genes/proteins [18]. Obtained cytokines genes were uploaded onto STRING database to obtain the relationships of protein–protein interaction (PPI), such as co-expression and co-localization. Finally, Cytoscape 3.3.0 software was used to construct PPI network.

### Molecular docking

To obtain a deeper understand about the association of quercetin with TNF-α and IL-1β, molecular docking was applied to evaluate the strength and mode of interactions between quercetin and TNF-α/IL-1β. The crystal structure of TNF-α and IL-1β were obtained from RCSB Protein Data Bank (PDB, http://www.rcsb.org/). ChemDraw software or PubChem (https://pubchem.ncbi.nlm.nih.gov/) was used to prepare the chemical structure of quercetin. Autodock 4.2 (http://mgltools.scripps.edu/downloads) and Discovery Studio software were applied for molecular docking.

## Results

### Identification of bioactive components of *R. crenulata*

We searched for *R. crenulata* by retrieving the TCMSP database and previous studies, and found that four active ingredients of *R. crenulata* with OB ≥30 % and DL index ≥0.18 were mainly consisted of quercetin (MOL000098), kaempferol (MOL000422), kaempferol-3-O-α-l-rhamnoside (MOL012777) and tamarixetin (MOL004083, Table 1).

**Table 1.** Information for bioactive components of Hongjingtian

Molecule ID	Molecule name	MW	OB (%)	DL	BBB	HL	TPSA	RBN
MOL000098	quercetin	302.25	46.43	0.28	−0.77	14.40	131.36	1
MOL000422	kaempferol	586.25	41.88	0.24	−0.55	14.74	111.13	1
MOL012777	kaempferol-3-O-α-l-rhamnoside	434.43	41.88	0.69	−1.65	16.15	166.14	3
MOL004083	tamarixetin	316.28	32.86	0.31	−0.44	14.59	120.36	2

MW, molecular weight; OB, oral bioavailability; DL, drug-likeness; BBB, blood-brain barrier; HL, Drug half-life; PSA, polar surface area; RBN, rotatable bond number.

### Construction of *R. crenulata*-compound-target network

**Fig. 1.**
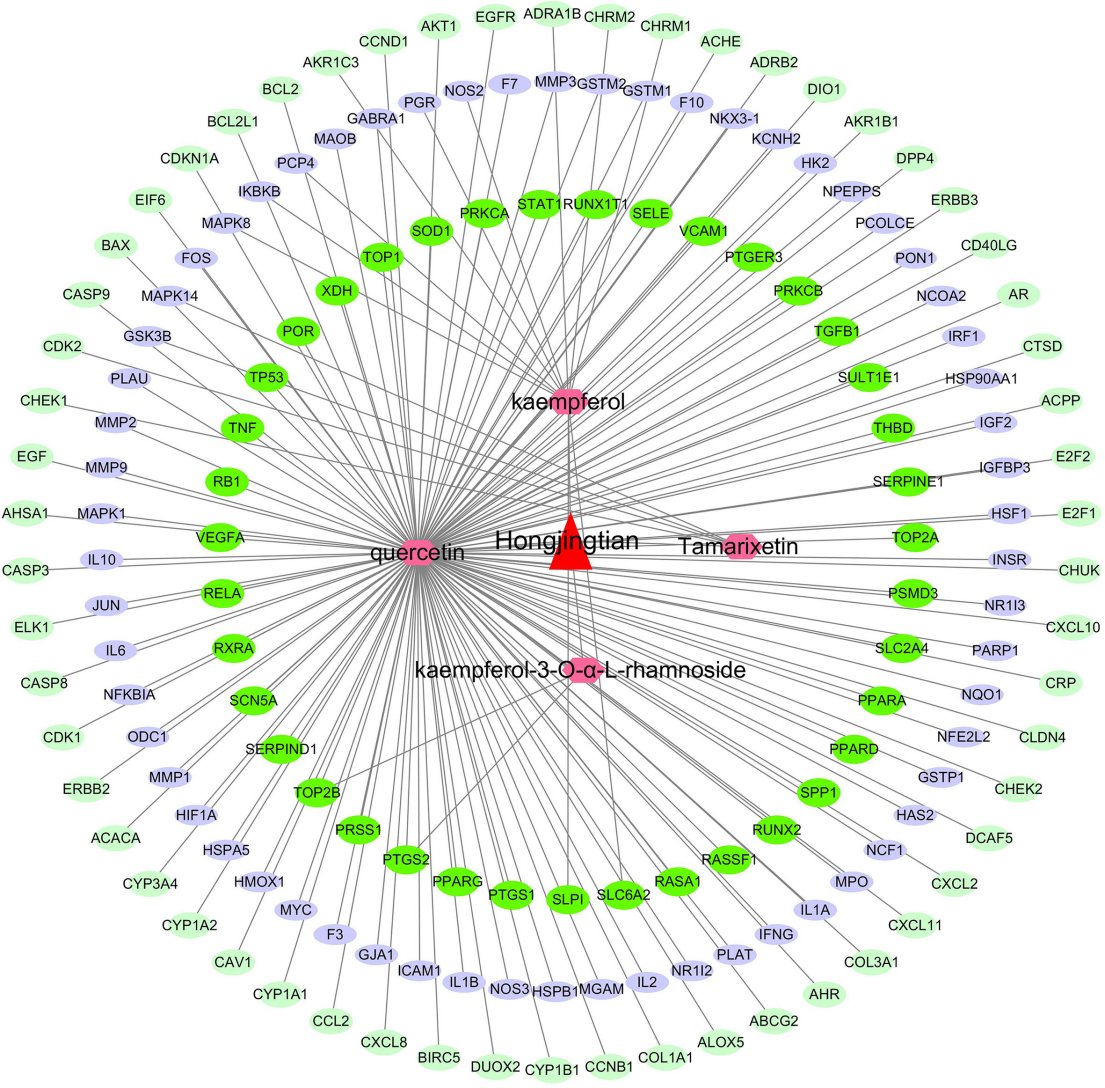
*R. crenulata* active compounds' putative targets' network diagram. The triangle in the figure represents the *R. crenulata*, the hexagons represents the active compound and the ovals represent the targets.

The target proteins of the effective components were obtained from the TCMSP database. Finally, 159 potential targets (without repetition) of four bioactive components were collected (Table 2). The pharmacological effect of herbs in preventing and controlling complex diseases might be associated with the synergy between multiple compounds and their targets. Here, *R. crenulata*-compound-target network was constructed (Fig. 1), which included 164 nodes (one for *R. crenulata*, four for candidate bioactive components and 159 for potential protein targets) and 165 edges. There were these components associated with targets, namely, quercetin (degree=144), kaempferol (degree=11), kaempferol-3-O-α-l-rhamnoside (degree=2) and tamarixetin (degree=4).

**Table 2.** Basic information list of chemical constituents in Hongjingtian

Protein	UniProt	Protein	UniProt	Protein	UniProt	Protein	UniProt	Protein	UniProt
EIF6	P56537	NQO1	P15559	VEGFA	P15692	PPARA	Q07869	PON1	P27169
CYP3A4	P08684	INSR	P06213	MGAM	O43451	PPARD	Q03181	DIO1	P49895
PTGS2	P35354	IL1A	P01583	HSPB1	P04792	CLDN4	O14493	PARP1	P09874
SLC6A2	P23975	EGFR	P00533	MAPK8	P45983	DUOX2	Q9NRD8	IKBKB	O14920
MMP9	P14780	F10	P00742	HAS2	Q92819	HSPA5	P11021	AHSA1	O95433
PTGS1	P23219	CYP1A1	P04798	COL1A1	P02452	SCN5A	Q14524	PLAU	P00749
MMP3	P08254	NCF1	P14598	NFE2L2	Q16236	RASA1	P20936	NOS3	P29474
CXCL11	O14625	RXRA	P19793	XDH	P47989	IL6	P05231	SULT1E1	P49888
MMP2	P08253	RELA	Q04206	ACHE	P22303	BIRC5	O15392	NOS2	P35228
IL10	P22301	F7	P08709	CAV1	Q03135	IGF2	P01344	TOP2B	Q02880
MMP1	P03956	NPEPPS	P55786	CYP1B1	Q16678	SELE	P16581	MYC	P01106
TGFB1	P01137	CYP1A2	P05177	CCL2	P13500	PRKCA	P17252	TOP2A	P11388
CXCL10	P02778	AHR	P35869	ERBB3	P21860	ICAM1	P05362	SLPI	P03973
GSTM1	P09488	POR	P16435	ERBB2	P04626	CDK1	P06493	KCNH2	Q12809
PGR	P06401	MAPK1	P28482	NFKBIA	P25963	AR	P10275	GSTP1	P09211
GSTM2	P28161	DCAF5	Q96JK2	ELK1	P19419	STAT1	P42224	TNF	P01375
TOP1	P11387	HIF1A	Q16665	CHEK1	O14757	SOD1	P00441	CXCL2	P19875
SLC2A4	P14672	THBD	P07204	CHEK2	O96017	CCNB1	P14635	COL3A1	P02461
SERPINE1	P05121	CD40LG	P29965	BCL2L1	Q07817	GSK3B	P49841	E2F2	Q14209
IFNG	P01579	F3	P13726	NKX3-1	Q99801	MAPK14	Q16539	CHUK	O15111
IL1B	P01584	JUN	P05412	EGF	P01133	RASSF1	Q9NS23	DPP4	P27487
RUNX2	Q13950	PPARG	P37231	PTGER3	P43115	IGFBP3	P17936	HK2	P52789
SPP1	P10451	CRP	P02741	HSP90AA1	P07900	CXCL8	P10145	PRSS1	P07477
PLAT	P00750	GJA1	P17302	RUNX1T1	Q06455	PCOLCE	Q15113	NR1I2	O75469
ODC1	P11926	AKT1	P31749	TP53	P04637	VCAM1	P19320	ADRA1B	P35368
GABRA1	P14867	AKR1C3	P42330	RB1	P06400	BCL2	P10415	CASP8	Q14790
MAOB	P27338	FOS	P01100	CDK2	P24941	BAX	Q07812	PSMD3	O43242
ACACA	Q13085	CASP3	P42574	PRKCB	P05771	AKR1B1	P15121	CHRM1	P11229
ACPP	P15309	HSF1	Q00613	ADRB2	P07550	IRF1	P10914	CTSD	P07339
ABCG2	Q9UNQ0	CASP9	P55211	CCND1	P24385	MPO	P05164	IL2	P60568
NR1I3	Q14994	PCP4	P48539	NCOA2	Q15596	ALOX5	P09917	E2F1	Q01094
CDKN1A	P38936	HMOX1	P09601	CHRM2	P08172	SERPIND1	P05546		

### Gene ontology and pathway-enrichment analysis for targets of *R. crenulata*

**Fig. 2.**
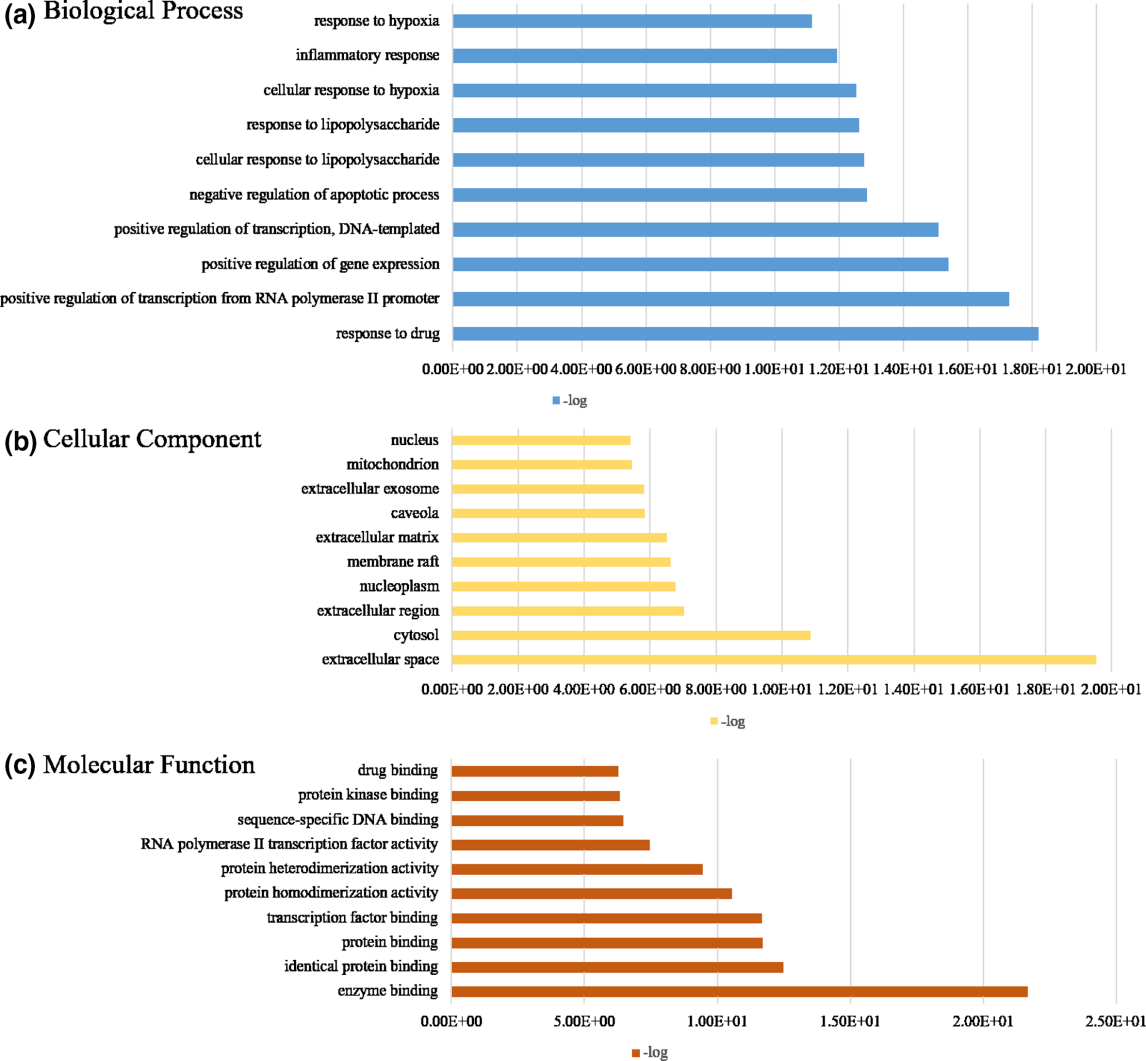
Gene ontology (GO) analysis of prescription targets. The *y*-axis shows significantly enriched (a) ‘biological process (BP)’ categories, (b) ‘cellular component (CC)’ categories and (c) ‘molecular function (MF)’ categories associated with the targets; the *x*-axis shows the enrichment scores (-logP) of these terms (top 10).

To recognize the potential biological functions of targets of *R. crenulata*, the GO annotation and pathway-enrichment analyses were conducted. There were respectively 463 biological process (BP), 47 cellular component (CC) and 98 molecular function (MF) terms in total (count of gene ≥2 and *P* value <0.05). Top ten significantly enriched BP, CC and MF categories were displayed in Fig. 2, Table 3. The possible BP were related to response to drug, positive regulation of transcription from RNA polymerase II promoter, positive regulation of gene expression, positive regulation of transcription DNA-templated, negative regulation of apoptotic process, cellular response to lipopolysaccharide, response to lipopolysaccharide, cellular response to hypoxia, inflammatory response and response to hypoxia (Fig. 2a). These genes were involved in CC including extracellular space, cytosol, extracellular region, nucleoplasm, membrane raft, extracellular matrix, caveola, extracellular exosome, mitochondrion and nucleus (Fig. 2b). Moreover, the MF of these genes were mainly correlated with enzyme binding, identical protein binding, protein binding, transcription factor binding, protein homodimerization activity, protein heterodimerization activity, RNA polymerase II transcription factor activity, sequence-specific DNA binding, protein kinase binding and drug binding (Fig. 2c).

**Table 3.** GO term for targets of Hongjingtian (top 10)

Term	Description	Count	P	-LOG(P)	Fold Enrichment	Bonferroni	Benjamini	FDR
Biological process (BP)								
GO:0042493	Response to drug	28	6.36E-19	1.82E+01	9.727243	1.46E-15	1.46E-15	1.11E-15
GO:0045944	Positive regulation of transcription from RNA polymerase II promoter	44	5.15E-18	1.73E+01	4.736843	1.19E-14	5.93E-15	9.01E-15
GO:0010628	Positive regulation of gene expression	24	3.97E-16	1.54E+01	9.674204	1.02E-12	3.40E-13	7.77E-13
GO:0045893	Positive regulation of transcription, DNA-templated	31	8.32E-16	1.51E+01	6.357111	1.79E-12	4.47E-13	1.35E-12
GO:0043066	Negative regulation of apoptotic process	27	1.35E-13	1.29E+01	6.266971	3.10E-10	6.20E-11	2.36E-10
GO:0071222	Cellular response to lipopolysaccharide	16	1.68E-13	1.28E+01	14.95364	3.85E-10	6.42E-11	2.93E-10
GO:0032496	Response to lipopolysaccharide	18	2.43E-13	1.26E+01	11.59135	5.58E-10	7.97E-11	4.24E-10
GO:0071456	Cellular response to hypoxia	15	2.93E-13	1.25E+01	16.50157	6.74E-10	8.42E-11	5.12E-10
GO:0006954	Inflammatory response	24	1.15E-12	1.19E+01	6.687708	2.65E-09	2.94E-10	2.01E-09
GO:0001666	Response to hypoxia	17	6.79E-12	1.12E+01	10.4382	1.56E-08	1.56E-09	1.19E-08
Cellular component (CC)								
GO:0005615	Extracellular space	52	2.88E-20	1.95E+01	4.424685	7.57E-18	7.57E-18	3.77E-17
GO:0005829	Cytosol	66	1.33E-11	1.09E+01	2.281955	3.50E-09	1.75E-09	1.74E-08
GO:0005576	Extracellular region	37	9.07E-08	7.04E+00	2.63404	2.39E-05	7.95E-06	1.19E-04
GO:0005654	Nucleoplasm	51	1.70E-07	6.77E+00	2.099653	4.48E-05	1.12E-05	2.23E-04
GO:0045121	Membrane raft	13	2.45E-07	6.61E+00	7.233071	6.46E-05	1.29E-05	3.21E-04
GO:0031012	Extracellular matrix	15	3.09E-07	6.51E+00	5.808261	8.12E-05	1.35E-05	4.04E-04
GO:0005901	Caveola	8	1.47E-06	5.83E+00	14.10663	3.87E-04	5.53E-05	0.001927
GO:0070062	Extracellular exosome	49	1.50E-06	5.82E+00	1.997937	3.95E-04	4.94E-05	0.001966
GO:0005739	Mitochondrion	30	3.47E-06	5.46E+00	2.583389	9.13E-04	1.02E-04	0.00455
GO:0005634	Nucleus	75	3.91E-06	5.41E+00	1.587484	0.001028	1.03E-04	0.005121
Molecular function (MF)								
GO:0019899	Enzyme binding	32	2.08E-22	2.17E+01	10.26708	1.03E-19	1.03E-19	2.99E-19
GO:0042802	Identical protein binding	33	3.25E-13	1.25E+01	4.707314	1.62E-10	8.09E-11	4.68E-10
GO:0005515	Protein binding	125	1.99E-12	1.17E+01	1.52023	9.90E-10	3.30E-10	2.87E-09
GO:0008134	Transcription factor binding	21	2.06E-12	1.17E+01	7.900272	1.02E-09	2.56E-10	2.96E-09
GO:0042803	Protein homodimerization activity	30	2.90E-11	1.05E+01	4.390758	1.44E-08	2.88E-09	4.17E-08
GO:0046982	Protein heterodimerization activity	23	3.66E-10	9.44E+00	5.284647	1.82E-07	3.04E-08	5.27E-07
GO:0004879	RNA polymerase II transcription factor activity, ligand-activated sequence-specific DNA binding	8	3.50E-08	7.46E+00	23.74262	1.74E-05	2.49E-06	5.04E-05
GO:0043565	Sequence-specific DNA binding	20	3.54E-07	6.45E+00	4.125165	1.76E-04	2.20E-05	5.10E-04
GO:0019901	Protein kinase binding	17	4.54E-07	6.34E+00	4.830612	2.25E-04	2.51E-05	6.53E-04
GO:0008144	Drug binding	9	5.17E-07	6.29E+00	12.65232	2.57E-04	2.57E-05	7.43E-04

**Fig. 3.**
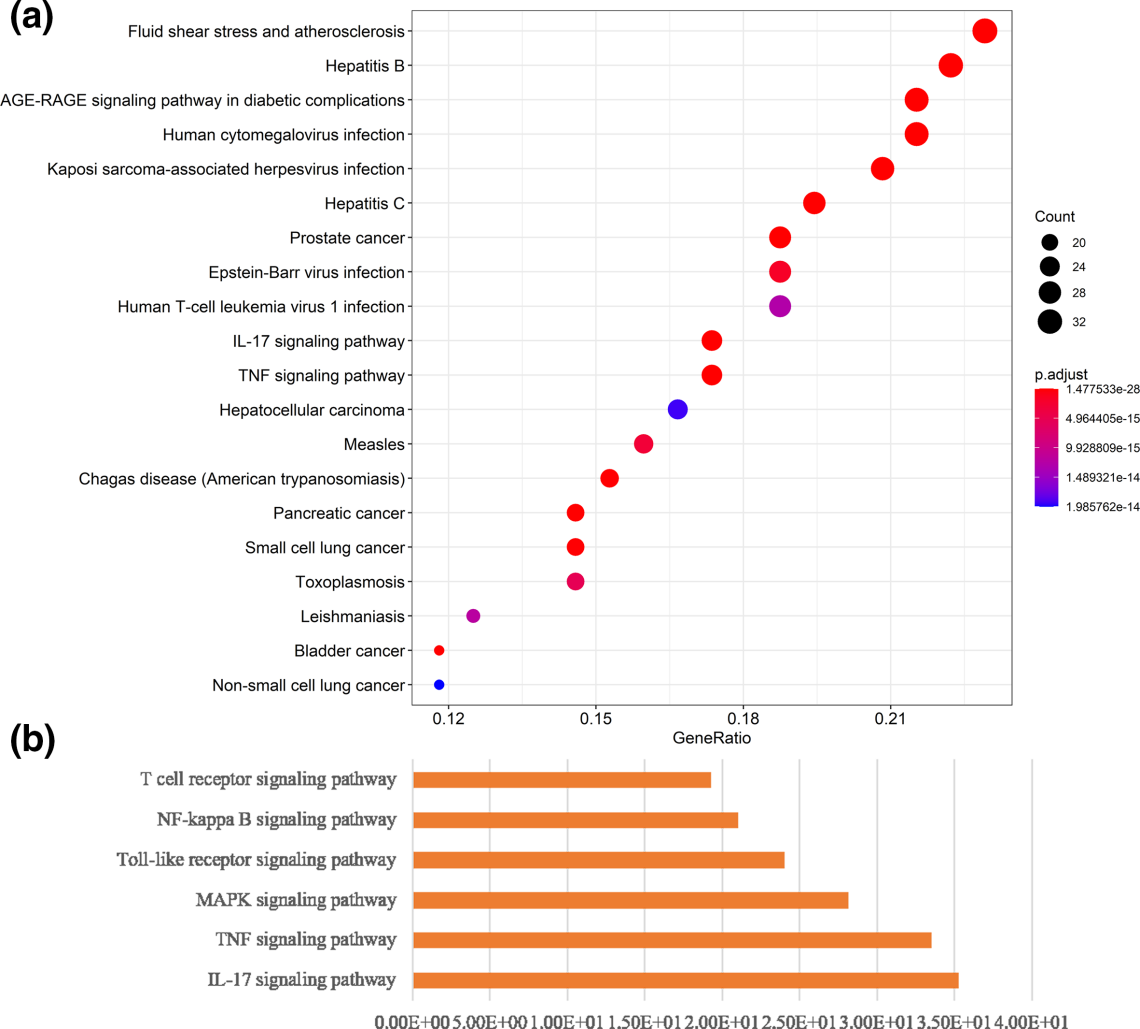
KEGG enrichment analysis diagram. The diagram of pathway-enrichment analysis the top 20 pathways (a) and immune-related signalling pathways (b) enriched by the KEGG method.

**Fig. 4.**
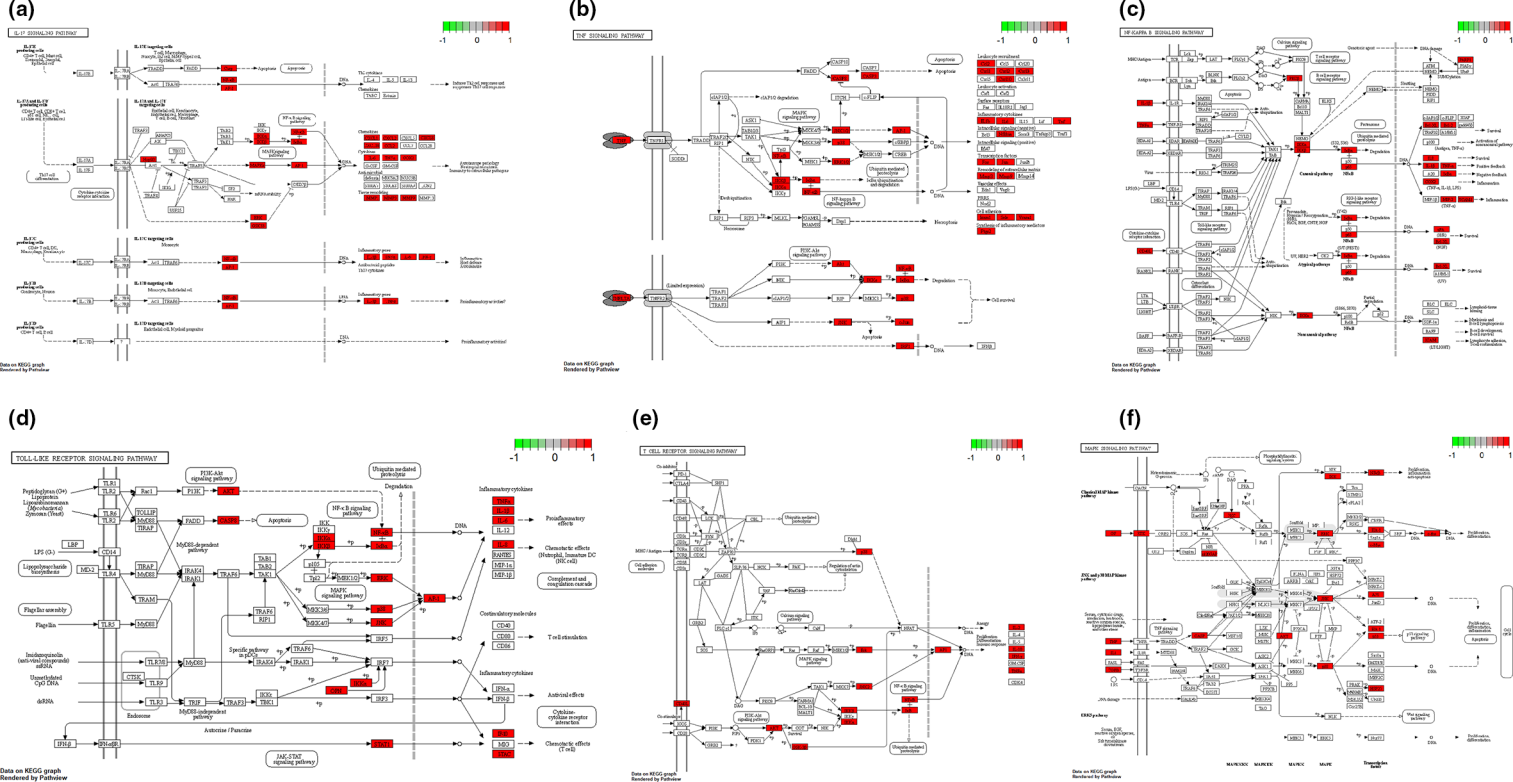
Diagram of immune-related signalling pathways. (a) IL-17 signalling pathway, (b) TNF signalling pathway, (c) NF-kappa B signalling pathway, (d) Toll-like receptor signalling pathway, (e) T cell receptor signalling pathway, (f) MAPK signalling pathway.

To determine the possible involved pathways of *R. crenulata* targets, KEGG-pathway analysis was performed. Top 20 enriched pathways of *R. crenulata* targets were shown in Fig. 3(a), Table 4. Moreover, KEGG-enrichment analysis showed that many target genes of *R. crenulata* were strongly associated with immune-related signalling pathways (Fig. 3b, Table S1, available in the online version of this article), including IL-17 signalling pathway, TNF signalling pathway, NF-kappa B signalling pathway, Toll-like receptor signalling pathway, T cell receptor signalling pathway and MAPK signalling pathway. KEGG diagram of immune-related signalling pathways were shown in Fig. 4.

**Table 4.** Annotation of pathways for targets of Hongjingtian (top 20)

ID	Term	Input no.	*P*-value	-LOG(P)	Corrected *P*-value
hsa05200	Pathways in cancer	60	3.31E-67	6.65E+01	8.15E-65
hsa04933	AGE-RAGE signalling pathway in diabetic complications	31	3.18E-46	4.55E+01	3.92E-44
hsa05418	Fluid shear stress and atherosclerosis	33	7.04E-46	4.52E+01	5.78E-44
hsa05161	Hepatitis B	32	3.83E-42	4.14E+01	2.35E-40
hsa05215	Prostate cancer	27	3.27E-39	3.85E+01	1.61E-37
hsa05167	Kaposi sarcoma-associated herpesvirus infection	30	3.24E-37	3.65E+01	1.33E-35
hsa05163	Human cytomegalovirus infection	31	1.60E-36	3.58E+01	5.62E-35
hsa04657	IL-17 signalling pathway	25	5.08E-36	3.53E+01	1.45E-34
hsa05160	Hepatitis C	28	5.31E-36	3.53E+01	1.45E-34
hsa04668	TNF signalling pathway	25	2.99E-34	3.35E+01	7.36E-33
hsa05169	Epstein–Barr virus infection	27	1.38E-31	3.09E+01	3.08E-30
hsa04151	PI3K-Akt signalling pathway	31	6.33E-31	3.02E+01	1.30E-29
hsa05212	Pancreatic cancer	21	1.06E-30	3.00E+01	2.00E-29
hsa05166	Human T-cell leukaemia virus 1 infection	27	1.14E-30	2.99E+01	2.01E-29
hsa05142	Chagas disease (American trypanosomiasis)	22	7.40E-30	2.91E+01	1.21E-28
hsa05222	Small cell lung cancer	21	5.48E-29	2.83E+01	8.42E-28
hsa05162	Measles	23	5.83E-29	2.82E+01	8.43E-28
hsa04010	MAPK signalling pathway	28	7.20E-29	2.81E+01	9.84E-28
hsa05225	Hepatocellular carcinoma	24	9.86E-29	2.80E+01	1.28E-27
hsa05219	Bladder cancer	17	1.76E-27	2.68E+01	2.16E-26

**Fig. 5.**
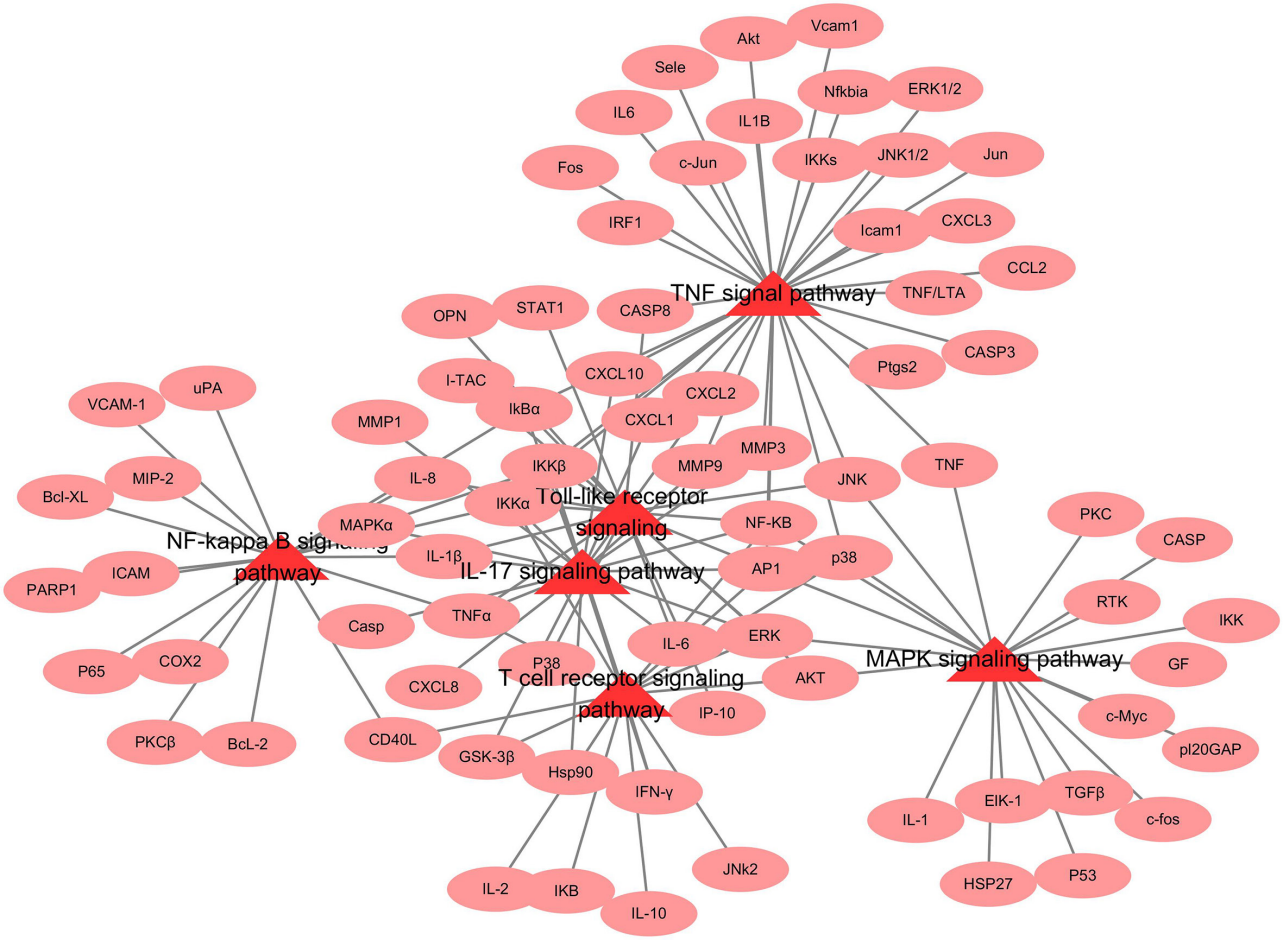
Targets-pathway network diagram. The triangle in the figure represents the immune-related signalling pathways, and the ovals represent the targets.

The illustrated network that a targets-pathway was established to understand their interaction (Fig. 5). Many targets were simultaneously involved in multiple biological processes. Among these potential targets, IkBα, TNF-α and IL-1β were identified as relatively high-degree targets, which played an essential role in NF-kappa B signalling pathway, Toll-like receptor signalling and IL-17 signalling pathway. The above results indicate that *R. crenulata* can exert an anti-inflammatory and immunoregulation through multiple targets and pathways.

### Protein–protein interaction analysis

**Fig. 6.**
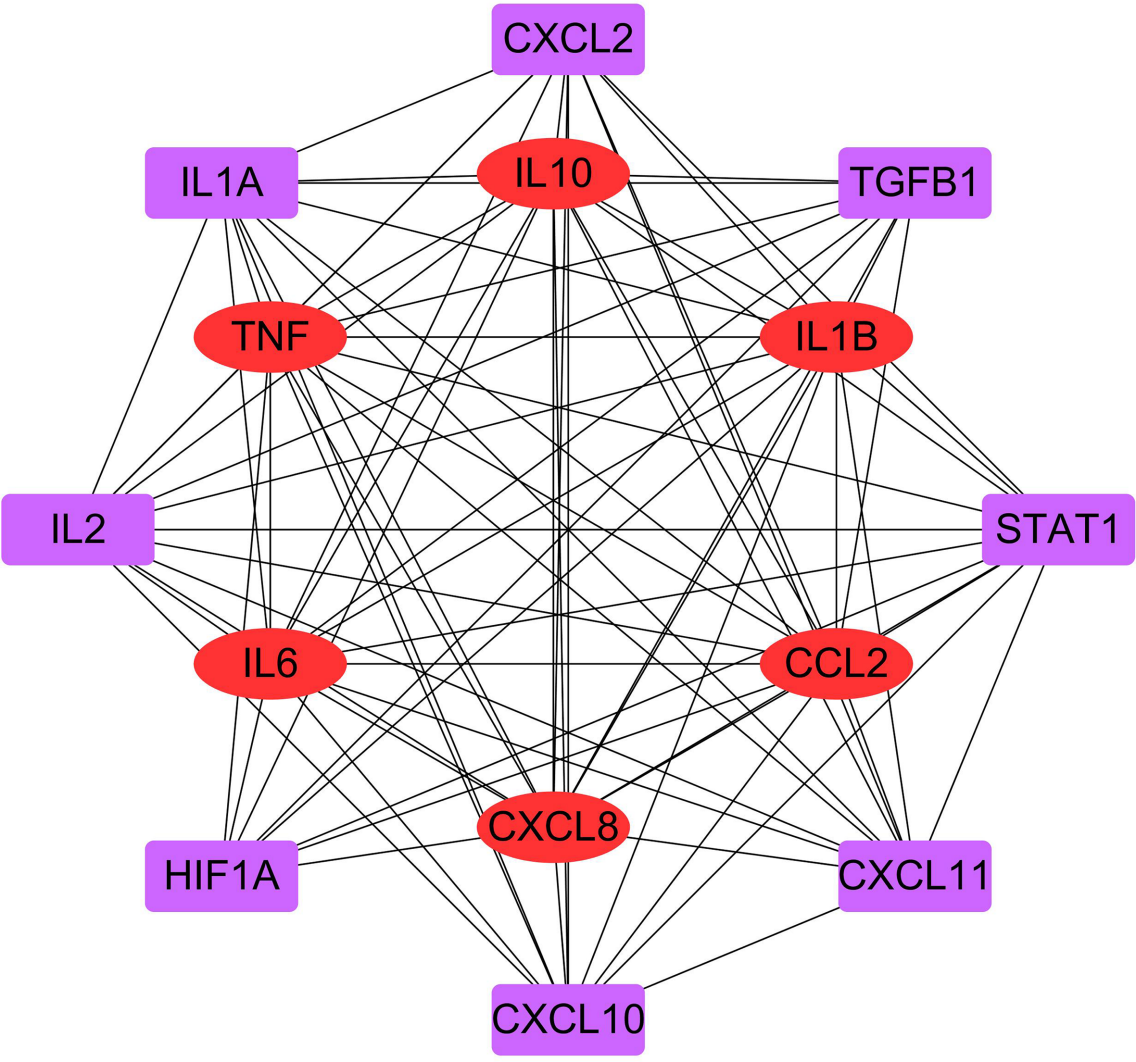
PPI network for the immunity and inflammation cytokines in COVID-19 among *R. crenulata* targets. The nodes represent proteins, and the connections represent interactions between proteins.

PPI network for the immunity and inflammation cytokines in COVID-19 among *R. crenulata* targets was displayed as Fig. 6. The results showed that IL10, IL-6, IL-1β, TNF-α, CCL2 and CXCL8 were important nodes in the network (Table S2).

### Molecular docking

**Fig. 7.**
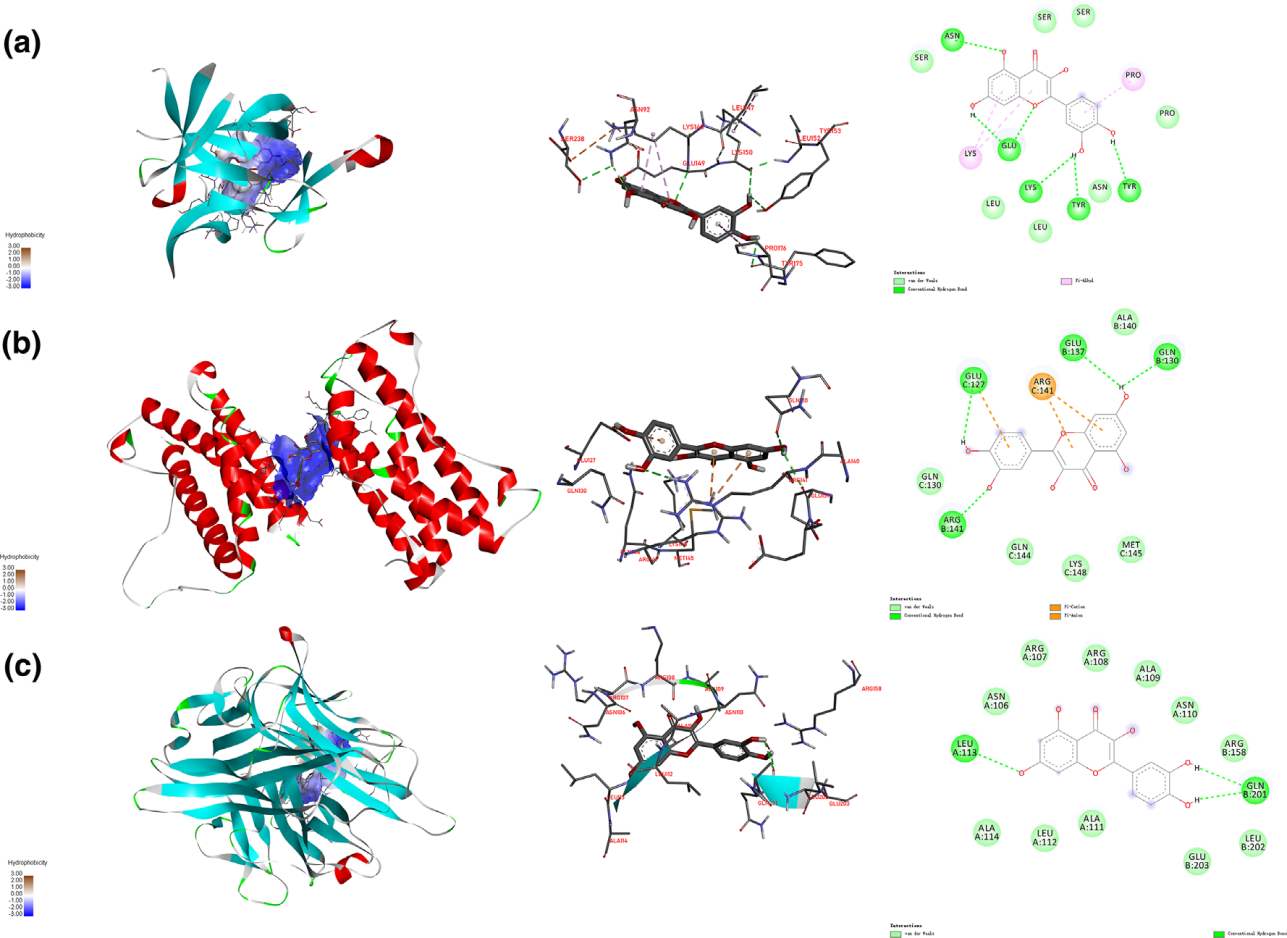
Molecular docking patterns of quercetin (MOL000098) with targets. (a) IL-6 protein-quercetin, (b) IL-1β protein- quercetin, (c) TNF-α protein-quercetin.

In COVID-19 patients, a pro-inflammatory status with high levels of IL-1β, IL-6 and TNF-α have been demonstrated [19]. In addition, IL-1β, IL-6 and TNF-α were important nodes in KEGG pathway and PPI network for *R. crenulata*. Here, molecular docking for *R. crenulata* ingredient (quercetin) and IL-1β, IL-6 and TNF-α proteins were analysed, and the results showed that quercetin had strong affinity with IL-1β, IL-6 and TNF-α proteins (Fig. 7). In terms of the interaction point, quercetin mainly interacted with amino acid residues ASN92, LYS148, GLU149, LYS150, TYR153, PRO176 and TYR175 of IL-1β (Fig. 7a). Quercetin and IL-6 formed a stable complex by interacting with the amino acid residues GLU127, ARG141, GLU137 and GLN130 (Fig. 7b). In addition, quercetin mainly interacted with amino acid residues LEU113 and GLN201 of TNF-α (Fig. 7c).

## Discussion

In this study, four bioactive components (quercetin, kaempferol, kaempferol-3-O-α-l-rhamnoside and tamarixetin) and 159 potential targets of *R. crenulata* were identified from the TCMSP database. *R. crenulata*-compound-target network diagram displayed the potential synergy between multiple compounds and their targets. Moreover, GO annotation and KEGG-pathway-enrichment analyses were conducted to recognize the potential biological functions of targets of *R. crenulata*. The result showed that target genes of *R. crenulata* were associated with inflammatory response and immune-related signalling pathways, including IL-17 signalling pathway, TNF signalling pathway, NF-kappa B signalling pathway, Toll-like receptor signalling pathway, T cell receptor signalling pathway and MAPK signalling pathway. Targets-pathway network and PPI network showed that IL-6, IL-1B and TNF-α were considered to be hub genes. Molecular docking showed that quercetin (MOL000098) had certain affinity with IL-1β, IL-6 and TNF-α. These results suggested that *R. crenulata* might play an anti-inflammatory and immunoregulatory role in the cytokine storm of COVID-19.

Hongjingtian (*R. crenulata*), a traditional Chinese medicine herb, can be mainly found at high altitudes in PR China such as Tibet, Qinghai. A recent study has revealed that the major bioactive components of *R. crenulata* have anti-inflammatory and antioxidant effects [20]. *R. crenulata* could achieve a certain effect on improvement of pulmonary inflammation of the mice infected with influenza virus and release of inflammatory factors in serum and lung tissue [11]. *R. crenulata* has the effect to improve the immunological functions of mice [6]. *R. crenulata* inhibit activation of NF-κB pathway to reduce acute lung injury caused by sepsis [21]. These studies support that *R. crenulata* has an important role in the inflammatory response. Recent studies have revealed that *R. crenulata* possess multiple active ingredients. Here, four active ingredients of *R. crenulata* with OB ≥30 % and DL index ≥0.18 were found including quercetin (MOL000098), kaempferol (MOL000422), kaempferol-3-O-α-l-rhamnoside (MOL012777) and tamarixetin (MOL004083). Moreover, 159 potential targets (without repetition) of four bioactive components were collected. *R. crenulata*-compound-target network diagram displayed that the potential synergy between multiple compounds and their targets.

GO annotation and pathway-enrichment analyses were conducted to recognize the potential biological functions for targets of *R. crenulata*. GO enrichment analysis displayed that the major biological processes included response to drug, positive regulation of transcription DNA-templated, negative regulation of apoptotic process, response to lipopolysaccharide, inflammatory response and response to hypoxia. KEGG-enrichment analysis showed that many target genes of *R. crenulata* were strongly associated with immune-related signalling pathways, including IL-17 signalling pathway, TNF signalling pathway, NF-kappa B signalling pathway, Toll-like receptor signalling pathway, T cell receptor signalling pathway and MAPK signalling pathway. IL-17 signals synergistically with numerous ligands that activate surprisingly diverse signalling pathways, such as TNF-α, IFN-γ, IL-13, or TGF-β [22]. A study reported targeting the IL-17 pathway to prevent acute respiratory distress syndrome associated with SARS-CoV-2 infection [23]. TNF signalling pathway has been identified as an important pathway in inflammatory response [24]. TNF induced the production of IL-6 and other cytokines, involved in the process of cytokine storm in COVID-19 [25]. NF-κ B/Nrf2 balance might be associated with the treatment of COVID-19 [26]. Toll-like receptor family members upregulated anti-viral and pro-inflammatory mediators (IL-6 and IL-8 and type I and type III interferons among others), through the activation of Nuclear Factor (NF)-kB in COVID-19 [27]. SARS-CoV infection could activate p38 MAPK and the downstream signalling possibly to increase human coronavirus viral replication leading to cell death [28]. These results indicate that *R. crenulata* may interfere with COVID-19 through multiple immune-related signalling pathways.

COVID-19 patients who succumb to pneumonia and hypoxia had one hallmark feature of the profound inflammatory state that marked elevation of serum inflammatory cytokines (IL-6, IFN-γ, IL-1β, TNF-α and TGF-β) and chemokines (CCL2, CCL5, CXCL8 and CXCL10) [29]. PPI network for the immunity and inflammation cytokines in COVID-19 among *R. crenulata* targets showed that IL-10, IL-6, IL-1B, TNF-α, CCL2 and CXCL8 were important nodes in the network. Combined with targets-pathway network, we found that IL-6, IL-1B and TNF-α were considered to be hub genes. In COVID-19 patients a pro-inflammatory status with high levels of IL-6, IL-1B and TNF-α has been demonstrated [19]. IL-6, a cytokine, has context-dependent pro- and anti-inflammatory properties, and excessive synthesis of IL-6 while fighting environmental stress leads to an acute severe systemic inflammatory response known as cytokine storm [30]. IL-1B, a member of IL-1 cytokine subfamily, has analgesic, immunomodulatory, anti-hypoxia and anti-inflammatory functions [31]. TNF-α regulates a variety of physiological functions in the body, including immune surveillance, immune response against microbial infections, and induction of cell death [32]. Subsequently, results of molecular docking indicated that quercetin (active ingredients of *R. crenulata*) could bind with IL-6, IL-1B and TNF-α. These results hinted that *R. crenulata* could regulate the formation of cytokine storms to reduce excessive inflammation in the body, thereby improving severe systemic damage in COVID-19 patients. However, the exact mechanism requires further validation in biological experiments.

## Conclusion

In summary, bioactive components and potential targets of *R. crenulata* were identified, and target genes of *R. crenulata* were associated with immune-related signalling pathways, especially IL-17 signalling pathway and TNF signalling pathway. Moreover, *R. crenulata* might play an anti-inflammatory and immunoregulatory role in the cytokine storm of COVID-19 by acting on IL-1β, IL-6 and TNF-α. However, further studies are necessary to elucidate the precise mechanism.

## Supplementary Data

Supplementary material 1
