# Supplementary material for: Network pharmacology and molecular docking analysis on mechanisms of Tibetan Hongjingtian (Rhodiola crenulata) in the treatment of COVID-19
Source: J Med Microbiol. 2021 Jul 27;70(7):001374. doi: 10.1099/jmm.0.001374 (PMC8493420; doi:10.1099/jmm.0.001374)
Supplement: Supplementary material 1 [file jmm-70-1374-s001.pdf]

Supplementary Table 1. Annotation of six immune-related signaling pathways for targets of Hongjingtian.

ID	#Term	Input number	P-Value	Corrected P-Value	Input
hsa04657	IL-17 signaling pathway	25	5.08E-36	1.45E-34	CHUK|CXCL2|HSP90AA1|PTGS2|CASP3|IKBKB|IFNG|CXCL8|CCL2|MMP1|MMP3|MMP9|GSK3B|CXCL10|JUN|TNF|NFKBIA|MAPK14|MAPK1|CASP8|MAPK8|IL6|FOS|RELA|IL1B
hsa04668	TNF signaling pathway	25	2.99E-34	7.36E-33	CHUK|CXCL2|PTGS2|IRF1|CASP3|ICAM1|IKBKB|VCAM1|CCL2|MMP3|AKT1|MMP9|CXCL10|JUN|TNF|NFKBIA|SELE|MAPK14|MAPK1|CASP8|MAPK8|IL6|FOS|RELA|IL1B
hsa04010	MAPK signaling pathway	28	7.20E-29	9.84E-28	CHUK|CASP3|IL1A|IKBKB|TP53|INSR|RASA1|TGFB1|AKT1|IGF2|EGFR|EGF|MYC|JUN|TNF|HSPB1|MAPK14|MAPK1|PRKCB|PRKCA|MAPK8|ELK1|VEGFA|FOS|RELA|ERBB2|ERBB3|IL1B
hsa04620	Toll-like receptor signaling pathway	19	9.21E-25	8.39E-24	RELA|IL6|MAPK14|CHUK|IKBKB|MAPK8|CXCL8|CXCL10|FOS|CASP8|STAT1|JUN|AKT1|IL1B|TNF|CXCL11|SPP1|NFKBIA|MAPK1
hsa04064	NF-kappa B signaling pathway	17	8.67E-22	5.08E-21	CD40LG|PRKCB|IL1B|ICAM1|IKBKB|BCL2|BCL2L1|CXCL8|VCAM1|PTGS2|NFKBIA|CXCL2|PARP1|TNF|RELA|PLAU|CHUK
hsa04660	T cell receptor signaling pathway	16	5.24E-20	2.63E-19	CD40LG|MAPK14|CHUK|IKBKB|MAPK8|IFNG|GSK3B|IL2|IL10|FOS|AKT1|JUN|TNF|RELA|NFKBIA|MAPK1

Supplementary Table 2. Fourteen important protein targets with top degree of PPI.

NO.	Drgree	Protein Target name
1	13	CCL2
2	13	CXCL8
3	13	IL1B
4	13	IL6
5	13	TNF
6	13	IL10
7	12	IL2
8	11	STAT1
9	11	CXCL2
10	11	CXCL11
11	11	CXCL10
12	11	IL1A
13	9	TGFB1
14	8	HIF1A
